# Salvage surgery after definitive chemoradiotherapy through VATS for an initial unresectable locally advanced lung cancer: an alternative consolidative modality to radiotherapy?

**DOI:** 10.1186/s40792-021-01227-2

**Published:** 2021-06-08

**Authors:** Hsuan-Hsuan Yu, Yi-Wei Chen, Yi-Chen Yeh, Chien-Sheng Huang, Chao-Hua Chiu

**Affiliations:** 1 Division of Thoracic Surgery, Department of Surgery, Taipei Veterans General Hospital, Taipei, Taiwan; 2 Divsion of Ratiotherapy, Department of Oncology, Taipei Veterans General Hospital, Taipei, Taiwan; 3 Department of Pathology, Taipei Veterans General Hospital, Taipei, Taiwan; 4 Division of Thoracic Oncology, Department of Chest Medicine, Taipei Veterans General Hospital, Taipei, Taiwan; 5 School of Medicine, National Yang-Ming Chiao Tung University, Taipei, Taiwan; 6 School of Medicine, Institute of Clinical Medicine, National Yang-Ming University, Section 2, Shih-Pai Road, 201 Taipei, Taiwan

**Keywords:** Non-small cell lung cancer, Definitive chemoradiotherapy, Salvage lung resection, Local consolidative treatment

## Abstract

**Background:**

Definitive chemoradiotherapy (dCRT) is the first choice treatment for patients with locally advanced non-small cell lung cancer (NSCLC), but up to 35% of dCRT-treated tumors may have persistent or recurrent disease. Since the last decades, multimodality therapy showing potential for cure has become the mainstream for treatment of locally advanced NSCLCs, even some that were initially inoperable. Although salvage lung resection after dCRT has been reported with acceptable survivals, experiences in this respect are still limited. Other concerns remain debatable and inconclusive, such as dosage of radiation exposure, long interval between dCRT and surgery, and surgical comorbidity.

**Case presentation:**

A 73-year-old male former smoker with diagnosis of right lower lobe of lung squamous cell carcinoma (SqCC) with multiple mediastinal lymphadenopathy, cT4N2M0, stage IIIB, received salvage right lower lobe + right middle lobe bilobectomy through video-assisted thoracoscopic surgery (VATS) after dCRT and adjuvant CRT to a total of 9000 cGy dosage of radiation. The interval from the 1st and 2nd ends of radiation to the salvage surgery was 980 and 164 days, respectively. The pre-operative forced expiratory volume in the first second was 2.33 L (101% predicted) and the diffusing capacity of the lungs for carbon monoxide was 56% predicted. The operating time was 6.5 h, and the total estimated blood loss was 50 ml. The patient was discharged on the 7th postoperative day without major complications or bronchopleural fistulas. The patient was still alive 42 months after the initial diagnosis of advanced N2 lung SqCC, and kept progression-free for 7 months after salvage lung resection.

**Conclusions:**

Salvage lung resection performed long after high-dose radiation therapy of dCRT is technically feasible through VATS approach in a patient with initially inoperable cT4N2M0 stage IIIB NSCLC, and can be an alternative consolidative treatment for locally advanced NSCLC.

## Background

According to NCCN guideline [1], patients diagnosed over stage IIIA or stage IIIB are not favored for surgery. Definitive concurrent chemoradiation therapy (dCRT) with high-dose radiation is offered as curative intent for tumors originally considered inoperable. However, dCRT alone as a curative treatment was associated with poor overall survival (OS) and high incidence of local failure [2, 3].

The possibility of persistent or recurrent local disease after dCRT has raised the discussion of lung resection after dCRT as a chance for improving long-term survival. Salvage lung resection is thus defined as the surgery performed beyond 90 days after high-dose dCRT for curative intent in patients with no initial plan for surgery [4, 5].

Salvage surgery may confront technical difficulties during operation, such as unclear tissue planes in the field operated on due to post-radiation fibrosis, to result in higher surgical complication rate and compromised wound healing [6]. Tissue ischemia after lung tissue radiation may also increase the risk of post-resection bronchopleural fistulas (BPF) [4]. Thus, the issue of salvage lung resection is still under debate.

Herein we reported a case who underwent video-assisted thoracoscopic surgery (VATS) as salvage lung resection after dCRT and adjuvant CRT with high-dose radiation therapy to a total of 9000 cGy. The long interval between radiotherapy (RT) and salvage surgery indicated a probably higher risk of tissue ischemia and fibrosis in operated field. The minimal invasive approach also posed a challenge for salvage lung resection with the possibility of tissue adhesion after RT.

## Case presentation

**Fig. 1:**
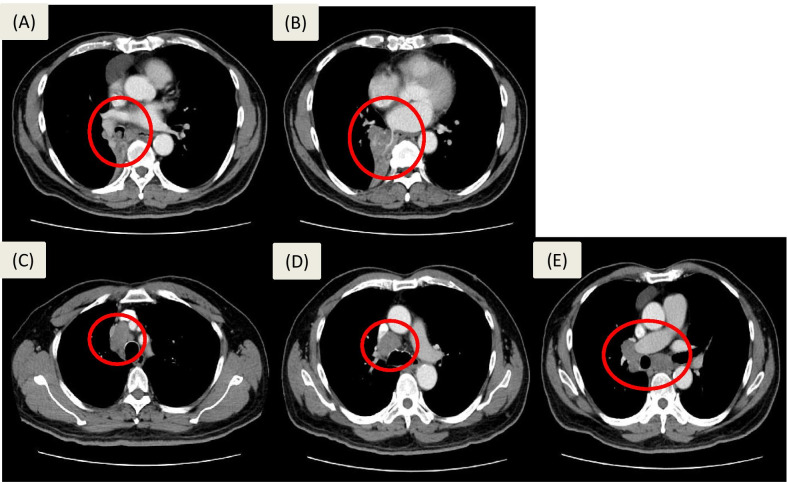
Series of chest CT scan images before treatment showing RLL lung tumor, cT4N2M0, stage IIIB, invading into the RIMB and right inferior pulmonary vein (**a**, **b**). Enlarged lymph nodes were noted at right para-tracheal, pre-carina, right hilar and sub-carina regions, compatible with metastatic lymphadenopathy (**c**, **d**, **e**). A cystic anterior mediastinal lesion was also noted

A 73-year-old man, a heavy smoker since age 18, had quit smoking for 10 years. He had severe and intermittent cough with whitish sputum and general malaise since 2017. Initial chest computed-tomography (CT) on 2017/10/16 showed irregular shaped consolidative tumor measuring 3.8 cm in the right lower lobe (RLL) with atelectasis, and invading to the right intermediate bronchus (RIMB) and right inferior pulmonary vein. Enlarged lymph nodes were noted at right para-tracheal, pre-carina, right hilar and sub-carina regions, compatible with metastatic lymphadenopathy (Fig. 1). Pathologic reports for the main tumor via bronchoscopic biopsy on 2017/11/09 reported squamous cell carcinoma (SqCC). Brain magnetic resonance imaging (MRI) and whole body bone scan in 2017/11 showed no brain and bone metastasis.

Treatment with dCRT was then suggested under the diagnosis of RLL lung SqCC with multiple mediastinal lymphadenopathy, cT4N2M0, stage IIIB. Chemotherapy was administrated with weekly cisplatin (Kemoplat®) 52.2 mg and oral vinorelbine (Navelbine®) 100 mg from 2017/12/11 to 2018/02/12, and RT was administrated with 6000 cGy in 30 fractions from 2017/12/19 to 2018/01/30.

**Fig. 2:**
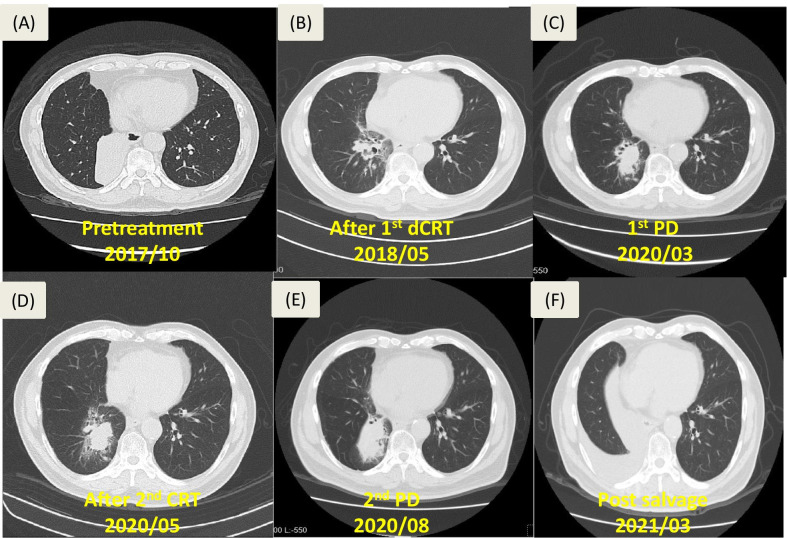
Series of chest CT scan images showing the tumor morphology from pre-treatment to pre-operative state. **a** Before 1st dCRT on 2017/10/16; **b** after the 1st dCRT on 2018/05/15; **c** post-dCRT surveillance showing tumor recurrence on 2020/03/04; **d** after the 2nd dCRT on 2020/05/18; **e**before salvage resection on 2020/08/18; **f** surveillance on 2021/03/22, 6 months after the surgery

However, the surveillance chest CT scan on 2020/03/04 demonstrated tumor progression with consolidation increasing to 4.5 cm. Restaging brain MRI and whole body bone in 2020/03 reported no brain and bone metastasis. Thus, a secondary course of chemotherapy with weekly cisplatin 54 mg and oral vinorelbine 100 mg was arranged from 2020/04/06 to 2020/04/27, along with a secondary course of RT with 3000 cGy in 15 fractions from 2020/04/06 to 2020/04/24. However, follow-up chest CT on 2020/08/18 still showed a continuing increase of the lesion to 4.7 × 5.1 cm (Fig. 2).

**Fig. 3:**
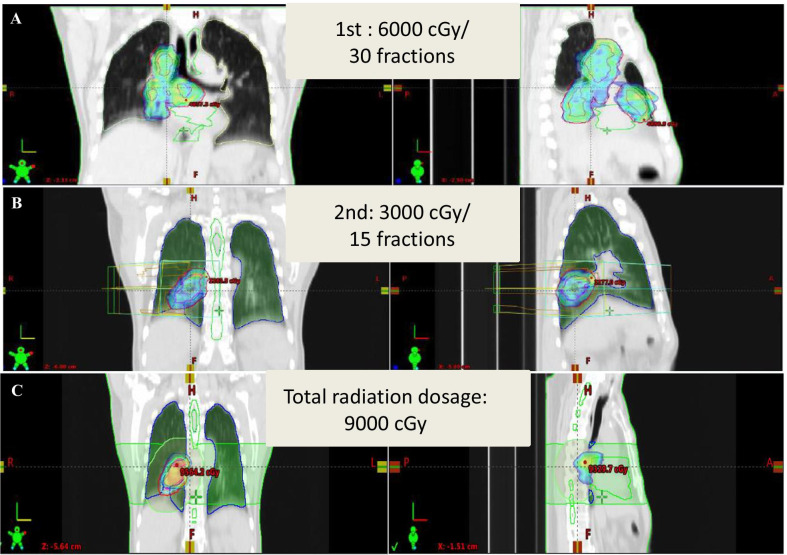
Definitive radiotherapy with radical intent was delivered. **a** The 1st R/T: 6000 cGy/ 30 fractions for gross tumor volume (GTV) onto the primary tumor over the RLL + grossly mediastinal LAPs; **b** the 2nd R/T: 3000 cGy/ 15 fractions for gross tumor volume onto RLL lung tumor; **c** total radiation dosage: 9000 cGy

**Fig. 4:**
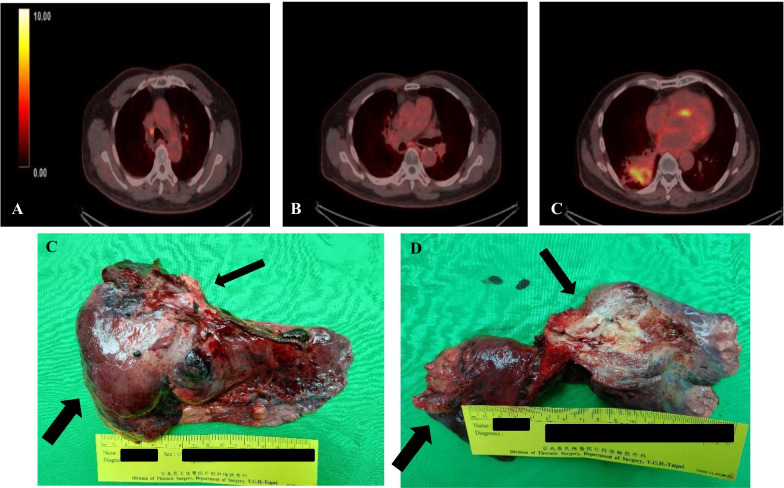
The preoperative whole body PET demonstrated residual tumor and metastatic lymphadenopathy. **a** Node with increased uptake in the right lower paratrachea (SUVmax 6.68); **b** another nodule with increased uptake in subcarina (SUVmax 3.96); **c** the main tumor (4.3 × 3.1 cm, a consolidative mass) with increased uptake (SUVmax 8.03) in the RLL of lung; **d** surgical specimen of bilobectomy; thin black arrow: simultaneous stapling of PA and bronchus; wide black arrow: RLL tumor mass; E after cutting, the tumor was buried in RLL, about 7 × 5 × 4cm in size (black arrow); wide black arrow: resected RML

The multidisciplinary lung cancer meeting held afterward suggested surgical consolidative therapy as an alternative treatment modality to be undertaken without any further radiological consolidative therapy, in view that overall 9,000 cGy radiation dose had been delivered to the tumor fields (Fig. 3). Thus, the patient was restaged and whole body positron emission tomography (PET) scan on 2020/09/03 reported recurrent tumor (SUVmax: 8.03) and suspicious metastatic lymphadenopathy in the right lower para-trachea (SUVmax: 6.68) and sub-carina (SUVmax: 3.96) (Fig. 4). Brain MRI on 2020/10/03 reported no evidence of metastasis. The patient was restaged as ycT3N2M0, stage IIIB.

**Fig. 5:**
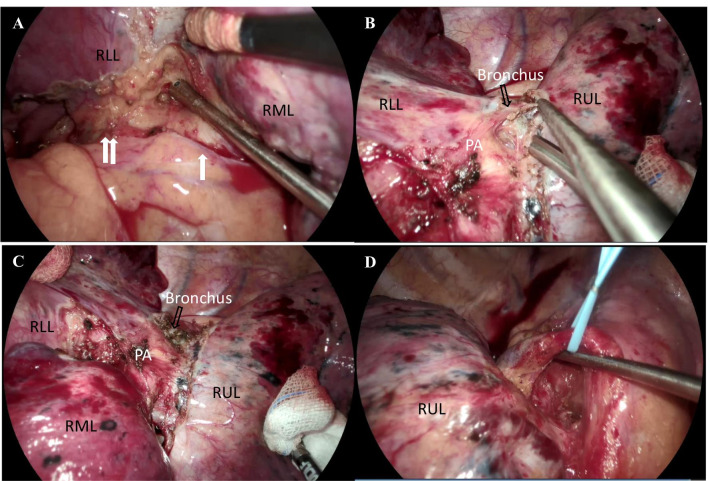
Intraoperative photographs: **a** RML PV (white arrow) and IPV (double white arrow); **b**, **c** dense plane between interlobar PA and RIMB (prior to simultaneous stapling); **d** No. 2 and No. 4 lymph node dissection

After admission, pre-operative FEV1 was 2.33L (101%) and DLCO was 56% predicted. Then salvage with video-assisted thoracic surgery (VATS) of RML + RLL bilobectomy was arranged on 2020/10/06 after a family meeting. Under double lumen anesthesia, a 5.0-cm incision was made over right 5th ICS in the anterior axillary line and wound protector was applied. Another 1.5 cm wound was made at 8th ICS, and thoracoport was inserted. Hilum was released first, and inferior pulmonary vein (IPV) and RML pulmonary vein (PV) were dissected. IPV and RML PV were divided first. The fibrotic change of the plane between intermediate pulmonary artery and RIMB made it difficult to separate division between artery and bronchus. Minor fissure was dissected first, and intermediate pulmonary artery and RIMB were divided by black-staple (Endo GIA Tri-Staple™ 45 mm, Covidien, USA). Radical lymph node dissection was performed including the subcarinal, interlobar, hilar, upper para-tracheal, and lower para-tracheal stations. Bleeding and air-leak were checked, and TISSEEL (4 ml; Baxter AG, Vienna, Austria) was used in the RIMB stump and para-tracheal lymph node region without other bronchial stump buttressing. One 24 French straight chest tube was placed via 8th ICS wound toward apex. Wound was closed in layers. During the operation, a pulmonary tumor about 7 × 5 × 4 cm in the right lower lobe attached to RIMB suspicious of airway invasion was resected (Figs. 4, 5). There was no pleural adhesion or seeding lesion, and mild serum-like effusion was noted. Estimated blood loss was 50 mL, and total operation time was 390 min.

The endotracheal tube was removed before the patient was transferred to postoperative room (POR). After checked with chest X-ray (CXR) under nasal canula 3 L/min, he was then transferred to respiratory intensive-care-unit (RICU) for intensive care. Mild subcutaneous emphysema was noted. No dyspnea or desaturation was noted under room air. The only complain about mild to moderate post-operative wound pain was relieved after adequate pain control with Dynastat® and Meitifen®. Follow-up CXR showed remaining subcutaneous emphysema without progression. The patient was transferred to ordinary ward on the postoperative 1st day. Chest tube was removed on the postoperative 5th day. Under stable clinical condition, he was discharged on the postoperative 7th day, and followed up at out-patient department. Post-salvage resection pathology showed SqCC of lung, keratinizing, 4.6 × 4.0 × 4.0 cm in size, with only 20% viable tumor cells, ypT1aN0, stage IA1. Follow-up chest CT done in 6 months revealed neither BPF nor tumor recurrence.

## Discussion

We reported a case of cT4N2M0 stage IIIB NSCLC, who was initially considered non-operable and received dCRT plus adjuvant CRT to the highest radiation dosage (6000 + 3000 cGy). Even with locoregional recurrence and long interval (980 days) between the 1st dCRT and salvage lung resection, VATS approach was successfully performed. To our knowledge, this case is unique and worthy of discussion and comparison with previous series, as summarized in Table 1.

**Table 1:** Literature review: salvage surgery after dCRT with high-dose radiation

Author /(published year /patient no.)	Yu et al*.* (2021/1)	Bauman (2008/24)	Kuzmik (2013/14)	Yang (2015/31)	Schreiner (2016/9)	Casiraghi (2017/35)	Romero (2019/27)	Bograd (2020/30)
Age (y/o)^a^	73	60	64	58	56.2	60–69	65.1	65.5
PreOP staging^b^	IIIB	IIIA	III	IIIA	IIIA	IIIA	IIIA/B	IIIA
Smoking history^b^	Former	N/A	N/A	Former	N/A	N/A	N/A	Former
Histology^b^	SqCC	NSCLC	NSCLC	NSCLC	NSCLC	NSCLC/SCLC	NSCLC	SqCC
Radiotherapy dose (cGy)^a^	9000	6390	5700	6000	6620	Mean 5800	6500	6000
dCRT to salvage OP (days)	164 (980)	147^a^	N/A	123.9^a^	211.4^a^	210^a^	250.6^a^	279ǂ
Pre-operative FEV1 (L)	2.33	2.3^a^	1.9^a^	69%^c^	N/A	N/A	N/A	N/A
Initially inoperable (No.)	1	8	2	8	1	14	12	8
Resection extent (Extended %)	Nil	20.8	28.6	19.4	78	45	N/A	37
Sublobar	–	–	3	–	1	–	–	–
Lobectomy	–	10	8	30	6	11	7	6
Bilobectomy	1	4	–	1	1	1	7	–
Sleeve lobectomy	–	–	1	–	–	–	–	–
Pneumonectomy	–	10	2	-	1	17	14	5
Exploratory only	–	–	–	–	–	6	–	–
Approach (open/VATS)	VATS	Open	Open	Open^b^	Open^b^	Open	Open	Open
Operating time (hour)^a^	6.5	5.5	N/A	N/A	3.9	N/A	N/A	4.9
Estimated blood loss (ml)^a^	50	250	N/A	N/A	400	N/A	N/A	150
Morbidity/mortality (%)	0	58.3(4.2)	43(0)	48(0)	22(11)	51(5.7)	14.8(3.7)	57(6.7)
Hospital stay after OP (days)^a^	8	8	10	4	20	7	8	7
Survival (months)^a^	42(7^d^)	3^d^	9^d^	32.5^d^	23^d^	13	75.6	24

*SqCC* squamous cell carcinoma, *ADC* adenocarcinoma, *N/A* not available, *SCLC* small-cell lung cancer

^a^Presented as median number

^b^Represented by the majority of patients included

^c^Presented as mean number

^d^Postoperatively

Up to date, experiences with salvage surgery after dCRT in lung cancer are still limited to report from each single institute [3, 4, 7–10]. High-dose radiation and long interval from dCRT to salvage surgery increase the risk of tissue ischemia and fibrosis in the operated field and subsequently increase the technical difficulty and operative comorbidities. In the study reported by Bograd et al., the median interval between dCRT and surgery was 279 days, and the post-operative complications rate was 57% [4]. As high as 8–13% of patients experienced major vascular injuries during surgery [4, 7]*.* In addition, Casiraghi et al*.* published a series of 35 patients who underwent salvage surgery after dCRT and reported 51% morbidity and 5.7% mortality rates. These results implied that long interval between dCRT and surgery did increase the technical difficulty in salvage lung resection. Accordingly, most of the salvage lung resections were done by traditional open method with proximal pulmonary artery control. However, with the continuous improvement of VATS equipment and surgical technique, it may turn technically safer, in some cases, for salvage lung resection by VATS after high-dose of radiation and long interval between dCRT and salvage surgery.

Relative fibrotic change between hilar planes was noted during operation. The crucial point for this operation lied in that the dissection and division for RML PV and IPV could be performed in advance. Additionally, the simultaneous stapling of PA and bronchus could be performed easily even in the presence of severe fibrotic change of the plane between intermediate pulmonary artery and RIMB. Together, before VATS becomes a standard approach for salvage lung surgery, surgical procedures that can guarantee safety and curability should be selected according to individual response after dCRT and surgeon’s experience.

One of the lethal postoperative complications resulting from high-dose of radiation and long interval between radiation and salvage surgery was BPF formation [6]. Accordingly, bronchial stump coverage using a vascularized flap was suggested in this group of patients who underwent routine surgical interventions [3–5, 9]. Even by doing that, the reported incidence of BPF was still around 3–7% and could be contributed to postoperative morbidity and mortality. In our case, we used tissue glue instead of vascularized flap and prevented from postoperative BPF. Fibrin sealant has been reported to treat small postoperative BPF [10]. However, this alternative method in prevention of BPF needs further evaluation in future studies. Another controversial issue is simultaneous stapling (pulmonary artery and bronchus) technique used in our patient. Bronchovascular fistula developed after salvage surgery is a catastrophic complication [3] but its’ association with simultaneous stapling remains undetermined. Even with the favorable report on the safety of simultaneous stapling from some Japanese study groups, we were not in a justified position to encourage its use in a general anatomic resection. However, on rare occasions, less invasive approach might be crucial as in our patient and echo our conclusion that, as an alternative consolidative modality to radiotherapy (after dCRT) as a salvage surgery, how extended surgery should we performed is still debatable [11].

The outcomes of salvage surgery for initially inoperable advanced stages of lung cancer address to another interesting issue. Although the current standard care for unresectable stage III NSCLC is dCRT followed by maintenance immune checkpoint inhibitor (ICI) therapy (it was not applied to our case because ICI was not yet approved locally at that time), there is currently no consensus on the effective local treatment strategy for patients with locoregional recurrence. Treatment options may include chemotherapy (± reirradiation), cryotherapy, radiofrequency ablation, observation only and/or salvage surgery [12]. In our case, as in the majority of clinical practices, salvage surgery was not taken into consideration to replace another course of CRT even when locoregional recurrence occurred after the 1st dCRT. The recurrent lesion continued to grow months after the 2nd course of CRT and consequently the decision of no more RT was made. Salvage surgery was then performed as an alternative consolidative treatment after consensus was obtained from the multidisciplinary meeting. In addition, the pathologic diagnosis showed only 20% of tumor cells survived, in contrast to progressive change of preoperative image (pseudo-progression phenomenon) [13]. Again, it should be emphasized that tissue assessment through primary lung tumor resection after systemic treatment of lung cancer is still the only strategy to reveal treatment effects currently even in the ear of advances in molecular target medicine and ICI therapy.

Satisfied survival has been reported in patients receiving dCRT followed by salvage surgery except extended resection [4, 11]. Interestingly, although longer interval between RT to salvage surgery may increase technical difficulty and morbidity, it was found to associate with better overall survival [3, 5]*.* Longer interval may possibly reflect the less aggressive nature of the tumor and act as a surrogate marker for patients who need salvage surgery. In contrast, non-surgical treatment for locoregional recurrence after dCRT treatment seemed to make poorer survival, 9–15 months, when compared with salvage surgery [5]. Currently, the observation period has been 7 months only after surgery; thus, further observation is needed to accurately discuss its prognosis.

## Conclusion

Our case demonstrated that salvage lung resection through VATS approach is feasible even long after the administration of high-dose RT after dCRT. Salvage surgery, even after two courses of CRT, should be considered as an alternative surgical consolidative modality for patients with persistent or local relapse disease. Further studies are required to identify effective patient selection and surgical criteria as favorable predictors of salvage surgery.

## Data Availability

Data sharing is not applicable to this article, as no datasets were generated or analyzed during the current study.

## Abbreviations

dCRT: Definitive chemoradiotherapy
SqCC: Squamous cell carcinoma
VATS: Video-assisted thoracoscopic surgery
OS: Overall survival
BPF: Bronchopleural fistula
CT: Computed-tomography
RLL: Right lower lobe
RIMB: Right intermediate bronchus
MRI: Magnetic resonance imaging
PET: Positron emission tomography
